# Serial prenatal and post-natal brain MRI demonstrates impact of congenital heart disease and cardiac surgery on brain growth and maturity

**DOI:** 10.1186/1532-429X-18-S1-P156

**Published:** 2016-01-27

**Authors:** Prashob Porayette, Jessie Mei Lim, Brahmdeep S Saini, Sujana Madathil, Meng Yuan Zhu, Edgar Jaeggi, Lars Grosse-Wortmann, Shi-Joon Yoo, Christopher Macgowan, Steven Miller, Mike Seed

**Affiliations:** 1 grid.42327.300000000404739646Paediatric Cardiology, The Hospital for Sick Children, Toronto, ON Canada; 2 grid.42327.300000000404739646Physiology & Experimental Medicine, The Hospital for Sick Children, Toronto, ON Canada; 3 grid.42327.300000000404739646Diagnostic Imaging, The Hospital for Sick Children, Toronto, ON Canada; 4 grid.42327.300000000404739646Neurology, The Hospital for Sick Children, Toronto, ON Canada

## Background

Fetuses and infants with congenital heart disease (CHD) have delayed brain maturation and lower brain volumes (BV) compared to normal [1-4]. To understand the impact of CHD and cardiac surgery on brain maturation, we performed serial brain MRI studies in patients with common cyanotic CHD before and after birth.

## Methods

Post-natal brain MRI were performed without sedation in 24 infants with common CHD before and after the cardiac surgery on a Siemens Avanto 1.5T system (Erlangen) after hospital IRB approval. 18 of 24 subjects also had fetal MRI using previously described technique [5] and BV and fetal weight were calculated [3]. The normal brain weights were obtained from published autopsy data [6] and converted to BV [7]. T2 mapping and diffusion weighted imaging were performed to measure T2 and apparent diffusion coefficient (ADC), respectively [2]. The mean T2 and ADC were measured in postnatal brains using 12 regions of interest located bilaterally at frontal and posterior white matter (WM) at inferior basal ganglia level; superior frontal and parietal WM at level of horns of lateral ventricles; and frontal and posterior centrum semiovale level. Cerebral oxygen delivery (CDO2) was also measured [1]. The daily change in BV, T2, and ADC were calculated by dividing the difference in values by days between the scans. The correlation between BV, T2, and ADC was examined using Pearson's Correlation.

## Results

**Figure 1:**
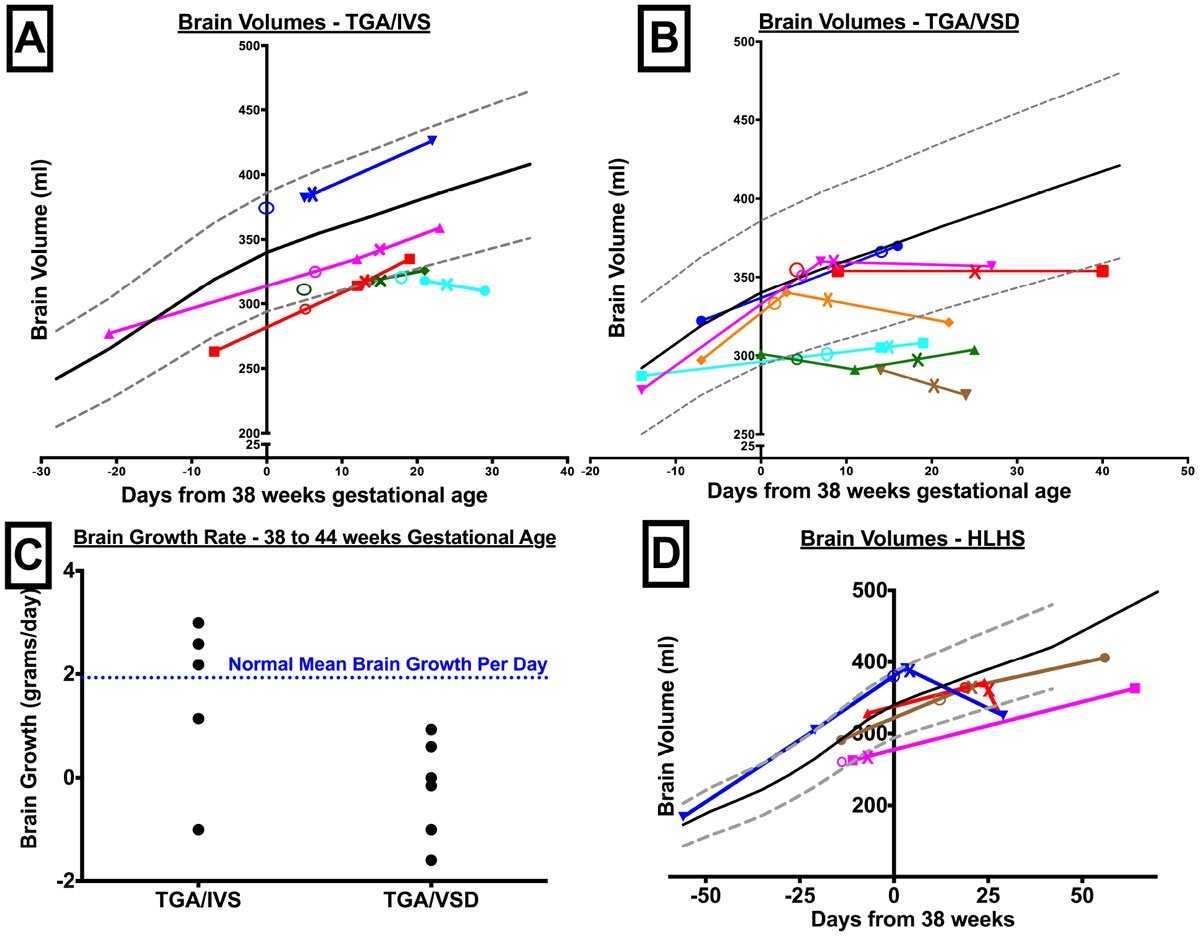
**Brain volumes in transposition of the great arteries with (TGA/IVS) or without intact ventricular septum (TGA/VSD) and hypoplastic left heart syndrome (HLHS)**. ‘0' on x-axis = 38 weeks gestational age (GA); black solid line: normal mean brain volume ± 1 SD (grey broken line) from autopsy series^6^; coloured lines: individual patient; ‘O': GA at birth; ‘X': time of surgery. (A) The brain in TGA/IVS continues to grow well after birth and surgery. (B) The brain growth in TGA/VSD plateaus or drops after birth and is not reverted immediately after surgery. (C) The TGA/VSD have lower brain growth rate than normals (blue line) during the 38 - 44 weeks GA. (D) The HLHS group showed similar decline in brain volume after cardiac surgery.

**Figure 2:**
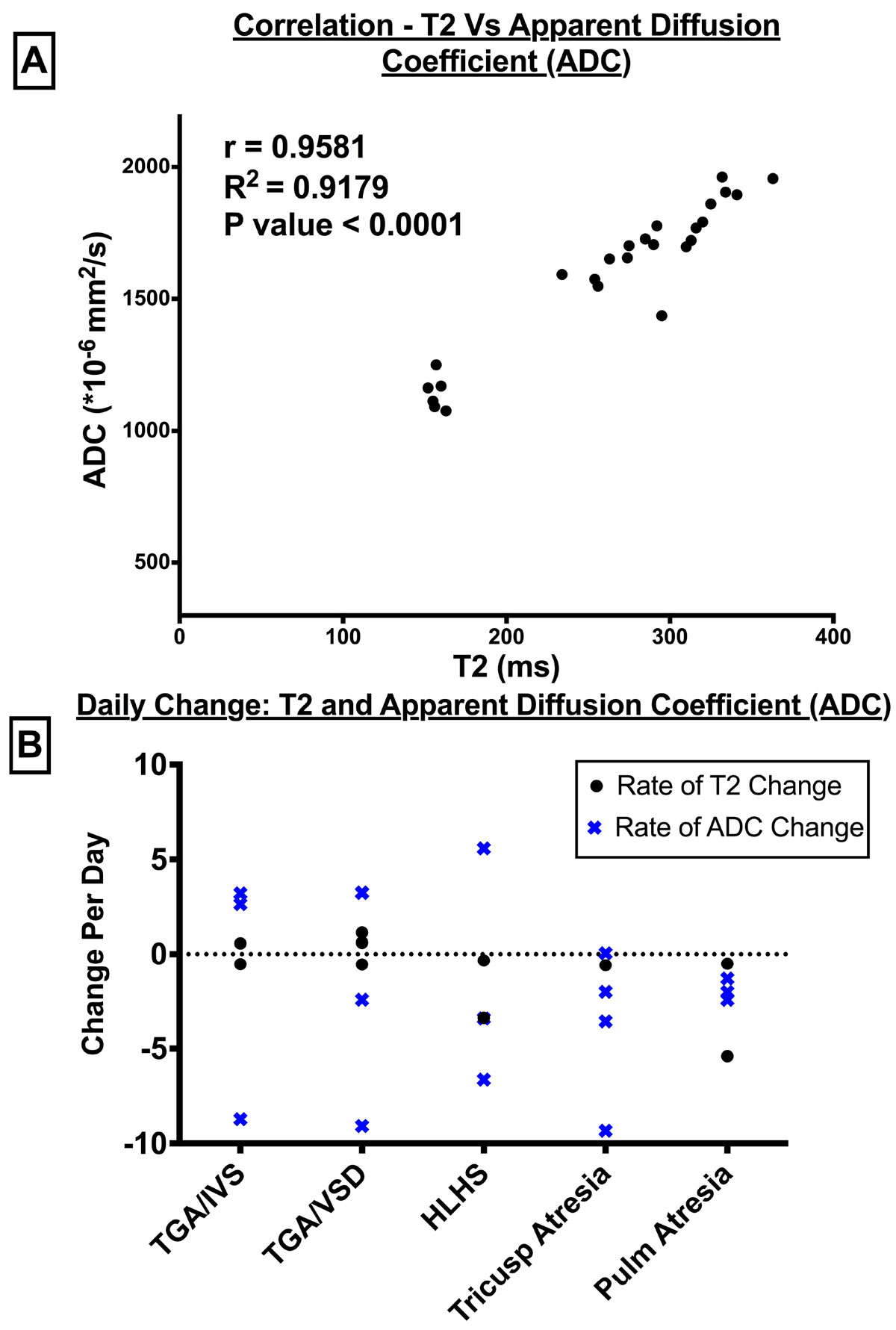
**Brain maturation in common cyanotic congenital heart disease**. (A) T2 and ADC values show high degree of correlation. T2 (r=-0.79, p < 0.0001) and ADC (r=-0.7, p < 0.0001) also correlated with brain volume. (B) T2 and ADC values decrease as the brain matures producing a net daily negative change. Children with TGA physiology have highest incidence of positive rate of change suggesting more immature brains among common CHD types.

The cohort (n = 24) consisted of patients with transposition of the great arteries (TGA) with intact ventricular septum (IVS; n = 5); TGA with ventricular septal defect (VSD; n = 7), hypoplastic left heart syndrome (HLHS; n = 4); tricuspid atresia (TA; n = 5), pulmonary atresia (PA, n = 3). The TGA/IVS group had normal brain growth after birth and surgery (Figure 1A). However, in TGA/VSD patients, the brain growth plateaus or drops after birth and do not revert immediately after surgery (Figure 1B). TGA/VSD had lower daily brain growth compared to normals (Figure 1C). HLHS showed similar decline in BV after surgery (Figure 1D). The infants with TA and PA had normal BV growth. The mean T2 and ADC values had excellent correlation (r = 0.96, p < 0.0001; Figure 2A). T2 (r=-0.79, p < 0.0001) and ADC (r=-0.7, p < 0.0001) also correlated with BV. Children with TGA physiology showed opposite change in T2 and ADC to expected values (Figure 2B). The mean CDO2/ml of brain was relatively lower in TGA/VSD (4 ml O2/min/ml BV; n = 3) compared to TGA/IVS (6 ml O2/min/ml BV; n = 3).

## Conclusions

Infants with TGA/VSD have the most immature brains among common cyanotic CHD probably related to low CDO2 *in utero* until surgery. Delayed repair leaves them exposed to adverse brain hemodynamics for a longer time. The reversal of normal decline in T2 and ADC in TGA indicates additional pathological process in these brains predisposing them to WM injury during cardiac surgery.
